# Single-cell clonal tracking of persistent T-cells in allogeneic hematopoietic stem cell transplantation

**DOI:** 10.3389/fimmu.2023.1114368

**Published:** 2023-02-10

**Authors:** Benedikt Obermayer, Luisa Keilholz, Thomas Conrad, Marco Frentsch, Igor-Wolfgang Blau, Lam Vuong, Stella Lesch, Kamran Movasshagi, Carola Tietze-Stolley, Lucie Loyal, Larissa Henze, Olaf Penack, Ulrik Stervbo, Nina Babel, Simon Haas, Dieter Beule, Lars Bullinger, Friedrich Wittenbecher, Il-Kang Na

**Affiliations:** ^1^ Core Unit Bioinformatics (CUBI), Berlin Institute of Health at Charite – Universitätsmedizin Berlin, Berlin, Germany; ^2^ Department of Hematology, Oncology, and Tumor Immunology, Charite – Universitätsmedizin Berlin, corporate member of Freie Universität Berlin and Humboldt-Universität zu Berlin, Berlin, Germany; ^3^ BIH Center for Regenerative Therapies (BCRT), Berlin Institute of Health at Charite – Universitätsmedizin Berlin, Berlin, Germany; ^4^ Core Unit Genomics, Berlin Institute of Health at Charite – Universitätsmedizin Berlin, Berlin, Germany; ^5^ Berlin Institute for Medical Systems Biology, Max Delbrück Center for Molecular Medicine in the Helmholtz Association, Berlin, Germany; ^6^ Stem Cell Facility, Charite - Universitätsmedizin Berlin, corporate member of Freie Universität Berlin and Humboldt-Universität zu Berlin, Berlin, Germany; ^7^ BIH Center for Exploratory Diagnostic Sciences (EDS), Berlin Institute of Health at Charite – Universitätsmedizin Berlin, Berlin, Germany; ^8^ Si-M/”Der Simulierte Mensch” a science framework of Technische Universität Berlin and Charite - Universitätsmedizin Berlin, Berlin, Germany; ^9^ Immunomics - Regenerative Immunology and Aging, Berlin Institute of Health at Charite – Universitätsmedizin Berlin, Berlin, Germany; ^10^ Center for Translational Medicine and Immune Diagnostics Laboratory, Medical Department I, Marien Hospital Herne, University Hospital of the Ruhr-University Bochum, Herne, Germany; ^11^ German Cancer Consortium (DKTK), Charite - Universitätsmedizin Berlin, corporate member of Freie Universität Berlin and Humboldt-Universität zu Berlin, Berlin, Germany; ^12^ ECRC Experimental and Clinical Research Center, Charite – Universitätsmedizin Berlin, corporate member of Freie Universität Berlin and Humboldt Universität zu Berlin, Berlin, Germany

**Keywords:** scRNAseq, allogeneic HSCT, leukemia, transplantation, T-cells, clonal tracking

## Abstract

The critical balance between intended and adverse effects in allogeneic hematopoietic stem cell transplantation (alloHSCT) depends on the fate of individual donor T-cells. To this end, we tracked αβT-cell clonotypes during stem cell mobilization treatment with granulocyte-colony stimulating factor (G-CSF) in healthy donors and for six months during immune reconstitution after transfer to transplant recipients. More than 250 αβT-cell clonotypes were tracked from donor to recipient. These clonotypes consisted almost exclusively of CD8^+^ effector memory T cells (CD8TEM), which exhibited a different transcriptional signature with enhanced effector and cytotoxic functions compared to other CD8TEM. Importantly, these distinct and persisting clonotypes could already be delineated in the donor. We confirmed these phenotypes on the protein level and their potential for selection from the graft. Thus, we identified a transcriptional signature associated with persistence and expansion of donor T-cell clonotypes after alloHSCT that may be exploited for personalized graft manipulation strategies in future studies.

## Introduction

Allogeneic hematopoietic stem cell transplantation (alloHSCT) is the standard of care with curative intent for various malignant and non-malignant hematological diseases (1, 2). In adult transplantation, stem cell grafts from peripheral blood (PB) of healthy donors after treatment with granulocyte-colony stimulating factor (G-CSF) are routinely used and currently the most prevalent graft source (1–3). Besides the mobilization of CD34^+^ hematopoietic stem cells into the periphery, G-CSF has direct effects on other immune cells (4–7) and leads to an increase in various immune cell types including several T-cell subsets such as CD8^+^ T-cells and regulatory T-cells (8–11). Clinical studies showed that transferred T-cells are critical for the success of alloHSCT as transplantations with T-cell depleted grafts have an inferior outcome (12, 13). Transplanted donor T-cells are pivotal in early immune protection and initial Graft-versus-Tumor effects (GvT), but on the downside, they may also cause Graft-versus-Host Disease (GvHD) (13, 14). GvHD is a possibly fatal complication that is mediated by alloreactive donor T-cells attacking host tissue. Together with immunosuppressive strategies, partial T-cell depletion *in vivo* by anti-thymocyte globulin (ATG), anti-lymphocyte globulin or attenuation of alloreactive T-cells by post-HSCT cyclophosphamide is used to reduce GvHD. However, this approach is limited as it thwarts essential GvT effects. Thus, finding the right balance of beneficial and adverse effects remains challenging. Development of strategies to optimize donor T-cells with anti-tumor activity are ongoing. Some of these strategies to optimize anti-tumor activity involve selective depletion of T-cells: The majority of human T-cells express the αβT-cell receptor (TCR) which endows these T-cells with the ability to recognize peptide antigens presented on HLA class I and II molecules. T-cells are largely separated into antigen experienced memory and naïve T-cells. Mouse studies show that transferred naïve T-cells are primarily responsible for GvHD, with memory T-cells causing only mild to no GvHD (15–21). Targeted depletion of naïve αβT-cells has been tested in clinical trials and resulted in very low incidences of severe acute GvHD or any grade of chronic GvHD, with no apparent increased risk of relapse or non-relapse mortality (22, 23).

In the context of these ongoing efforts and with the goal of further identifying persisting T-cell subsets and their associated phenotypes, we analyzed the fate of donor αβT-cell clonotypes and their transcriptional dynamics during G-CSF mobilization and in the posttransplant follow-up after alloHSCT. We used single-cell RNA sequencing (scRNAseq) enabling longitudinal analysis of transcriptional patterns of T-cell populations with unprecedented granularity. The integration of paired α and β chain TCR information on the single cell level furthermore makes it possible to assign exact clonal identity to single T-cells. In the context of alloHSCT, this means that for the first time we can track healthy donor T-cell clonotypes long-term after transfer to the transplant recipient and link distinct transcriptional attributes with clonal dynamics and persistence of T-cells.

## Methods

### Study design and approval

We designed our study to analyze peripheral blood lymphocytes of paired donor and recipient samples. We included five alloHSCT patients between December 2018 and May 2019 who received PB grafts from related donors at the Charité Universitätsmedizin Berlin. Patients were only included if the respective donors could be included as well. Blood samples were collected from donors before G-CSF mobilization and on the day of apheresis. Recipient samples were collected on days +90 and +180 post transplantation. This study was approved by the local ethics committee of Charité Universitätsmedizin Berlin (EA1/272/16) and all individuals gave informed consent.

### Sample preparation

PBMC were isolated from up to 20ml whole blood using density gradient centrifugation. PBMC were then freshly frozen according to standard procedures and stored in liquid nitrogen. All samples from one pair (i.e., donor samples from before and after G-CSF mobilization and recipient samples from days +90 and +180) were then treated in the same experimental run. The samples were thawed and stained with 4’,6- diamidino-2-phenylindole (DAPI). For all scRNAseq experiments, we sorted for alive lymphocytes using a FACSAria Fusion cell sorter (Becton Dickinson, Franklin Lakes, NJ, USA). Only for functional studies and TCR bulk sequencing experiments, sorting of CD8 T-cells was performed using a FACSMelody cell sorter (Becton Dickinson, Franklin Lakes, NJ, USA). Representative examples of the two different sorting strategies are shown in **Supplemental Figure S6**.

### Single-cell library construction and sequencing (CITEseq antibody labeling, scRNAseq, scTCRseq)

After cell-sorting we continued to treat samples individually, using one 10x lane per sample. Alive cells were incubated with 5 μl Human TruStain FcX™ per 1x10^6^ cells for 10 mins, then stained with nucleotide‐labeled TotalSeqC™ antibodies (Biolegend, San Diego, USA) for 30 min. To avoid antibody aggregates, labeled cells were washed 3 times with 1 ml PBS/BSA (BSA concentration 0.2%) with centrifugation at 300 g for 5 min. Cells were then resuspended in 50 μl PBS/BSA (BSA concentration 0.2%) and counted using the Neubauer chamber. We aimed for a calculated amount of ~16.500 cells of each sample for droplet encapsulation on separate lanes of the Chromium Controller (10x Genomics, Pleasanton, CA, USA). Single-cell capturing and library construction were performed with the Chromium Next GEM Single Cell V(D)J Reagent Kit v2 (10x Genomics, Pleasanton, CA, USA) according to manufacturer’s instructions. Essentially, single-cell gel beads-in-emulsion (GEMs) are formed, each containing a single cell and 10x chemistry for cell lysis, barcoding and reverse transcription of contained RNA within each GEM. The resulting cDNA including the single-cell barcodes is then amplified using standard polymerase-chain reaction (PCR). We constructed gene expression libraries with 10x 5’ Library Kits (PN-1000263/5, PN-1000190, PN-1000286, PN-1000215), and TCR libraries with the 10x T-cell V(D)J Enrichment Kits (PN-1000252). Sequencing of the resulting libraries was performed on an Illumina NovaSeq6000.

### Single-cell sequencing analysis

Sequencing libraries for gene expression and TCR/BCR were jointly processed using cellranger multi (v6.0.0) and the GRCh38 genome annotation, and analyzed with Seurat v4.0.11. We next used Seurat’s reference mapping workflow to jointly transfer celltype labels at different granularity (“levels”) and embedding coordinates from a PBMC reference (24), after filtering out cells with more than 10% mitochondrial gene content, less than 250 or more than 5000 genes and those with a level 1 cell type prediction score of less than 0.75. We used level 2 annotation for B and T-cells, and level 1 annotation otherwise. Next, we used scRepertoire v1.1.22 to process cellranger VDJ output. Persisting clonotypes (both chains) were defined as those appearing in at least one recipient and one donor sample each. Clonal diversity was assessed using the inverse Simpson score, and clonal overlap with the Morisita index. Antigen specificity was assessed using vdjmatch (v1.3.1) (25). Functional enrichment analysis was done with tmod v0.46.24 with gene sets from the Hallmark, Reactome, Kegg and Gene Ontology BP databases. We investigated differential cell-cell signaling between donors and recipients using scDiffCom v0.1.05. The cytotoxicity score was computed following Zhang et al. (26), i.e., by projecting our pseudobulk data onto the PCA space defined by their reference dataset (GSE124731). The effectorness score was computed analogously to the approach of Cano-Gamez et al. (27), i.e., by computing a pseudotime ordering of all CD8 T-cells with monocle3 v0.2.3.08 on the “integrated” assay obtained using Seurat’s Integrate Data workflow to remove batch variation between different samples (24). Computational enrichment of persisting cells from donor CD4^+^/CD8^+^ T cells was done with logistic regression and random forest classifiers (randomForest package v4.6-14). We first used 10fold cross-validation with a 75:25 train:test split across all cells to evaluate the classifiers and feature sets, and then another round of cross-validation, training on 3 donors and testing on the fourth.

### Statistical analysis

Differences in cell type composition were tested using mixed-effects binomial models (lme4 package, v1.1-27.1). Differential gene expression analysis was performed with DESeq2 v1.30.13 using a pseudo-bulk strategy, i.e., by summing up counts in all cells of the same type from the same sample, using pair identity as covariate. For functional experiments, we used paired t-tests in GraphPad Prism v9.4.1 (GraphPad Software, San Diego, CA, USA).

### TCRβ bulk sequencing and analysis

TCR repertoires were assessed as previously described by next-generation sequencing (28). Briefly, genomic DNA was isolated using AllPrep DNA Micro Kit (Qiagen) and the recombined V-CDR3-J region of the TCRβ locus was amplified. Purified amplicons were sequenced using Illumina HiSeq sequencing platform and clonotypes characterized using IMSEQ software (29).

## Results

### Clinical set-up for scRNAseq of PBMCs in alloHSCT

**Figure 1:**
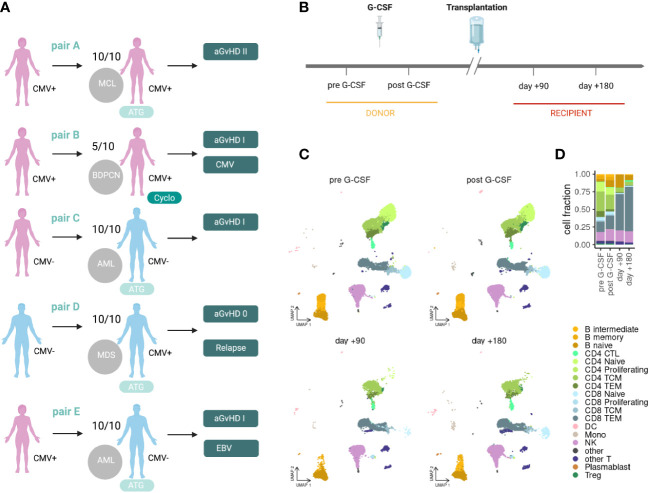
Clinical set-up for scRNAseq of PBMCs in alloHSCT. **(A)** Clinical characteristics of the included donor-recipient pairs. **(B)** Clinical set-up and sampling time points in the donor (pre G-CSF and post G-CSF) and recipient (day +90 and day +180). **(C)** scRNAseq data projected into a PBMC reference embedding for different time points before and after alloHSCT. **(D)** Stacked bar plot summarizing cell type fractions at different stages. MCL, mantle cell lymphoma; BPDCN, blastic plasmacytoid dendritic cell neoplasm; AML, acute myeloid lymphoma; MDS, myelodysplastic syndrome; 10/10, HLA-matched; 5/10, haploidentical; ATG, antithymocyte globuline; Cyclo, cyclophosphamide; aGvHD, acute graft-versus-host disease; CMV, cytomegalovirus; CTL, cytotoxic T lymphocytes; TCM, central memory T-cells; TEM, effector memory T-cells; DC, dendritic cells; Mono, monocytes; NK, natural killer cells; Treg, regulatory T-cells; pre, pre G-CSF; post, post G-CSF; d90, day +90 post transplantation; d180, day +180 post transplantation.

We performed massively parallel single-cell RNAseq and αβTCR profiling of peripheral blood mononuclear cells (PBMC) in donors and recipients of alloHSCT. We included five donor-recipient pairs (A-E) with four matched-related and one haploidentical transplantation. Clinical data are depicted in **Figure 1A** and summarized in **Supplementary Table 1**. All patients underwent myeloablative conditioning treatment. T-cell-depleting therapy with ATG was applied in the matched-related transplantations, and post-HSCT cyclophosphamide was applied in the haploidentical transplantation. Four of five recipients developed mild to moderate GvHD (overall score grade I-II (30)). One recipient required donor lymphocyte infusions due to relapse of disease with declining donor bone marrow chimerism starting at day+120 (pair D). There was one case each of mild cytomegalovirus (CMV) and Epstein-Barr virus (EBV) reactivation (pairs A and E, respectively). Samples were collected before and after G-CSF treatment in donors and in recipients on days +90 and +180 after transplantation (**Figure 1B**). In total, we sequenced 97,520 cells including 68,762 T-cells after quality control (**Supplementary Figure S1A**), with about 2,500 T-cells per sample and about 18,000 T-cells per time point (median). We used label transfer from a published multimodal PBMC cell type reference (24) to annotate different cell populations at different time points (**Figure 1C**). Further, we applied Cellular Indexing of Transcriptomes and Epitopes by sequencing (CITE-seq), a method combining multiplexed antibody-based detection of protein markers together with transcriptome profiling for single cells (31), and detected strong enrichment of antibody-derived tags for canonical markers in the associated immune populations (**Supplementary Figure S1B**).

### Donor-recipient shifts in cell composition, gene expression and intercellular signaling

**Figure 2:**
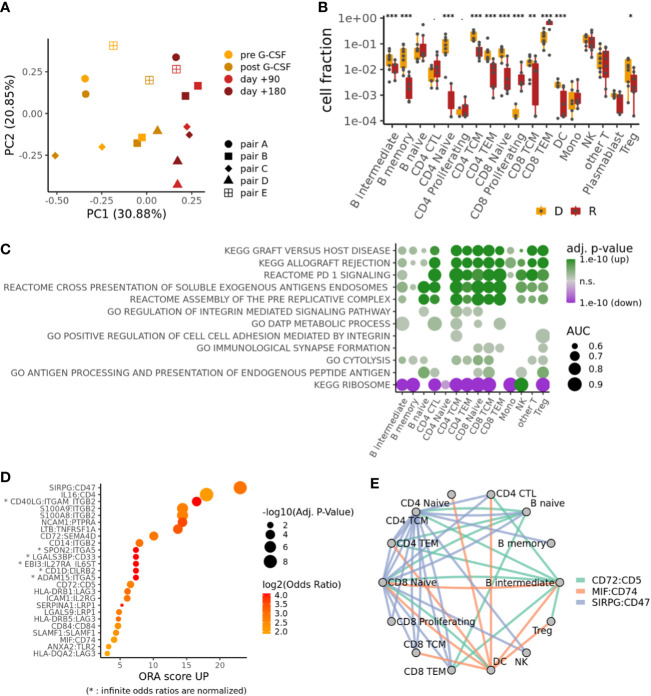
Donor-recipient shifts in cell composition, gene expression and intercellular signaling. **(A)** PCA on the cell type compositions of the different samples. **(B)** Compositional differences between samples from donors D (both pre and post G-CSF samples, if available) and recipients R (both d+90 and d+180 samples, if available). p-values from mixed-effects binomial model; ***p < 0.001, **p < 0.01, *p < 0.05. **(C)** Selected pathways differentially regulated between recipients and donors for each cell type; color indicates adjusted p-value and direction of change; size indicates effect size (area-under-curve). **(D)** Top ligand-receptor-interactions (LRIs) in an overrepresentation analysis (ORA) of differential cell-cell signaling between recipients and donors. **(E)** Cell-cell signaling network for top LRIs from **(D)**.

We analyzed all cells in our samples with a focus on differences between donors (healthy) and recipients (patients) and found a systematic shift in cell type composition between donors (pre G-CSF, post G-CSF) and recipients (day +90, day +180) (**Figures 1D**, **2A**; **Supplementary Figure S1C**). In particular, we observed an expansion of cytotoxic CD4^+^ T-cells (CD4 CTL) and proliferating and effector memory CD8^+^ T-cells (CD8TEM) at the expense of other populations including B-cells, dendritic cells (DC) and naive T-cells (**Figure 2B**). The CD8 T-cell expansion is based on large and hyperexpanded clonotypes (**Supplementary Figure S2A**). We further performed differential gene expression followed by gene set enrichment analysis between donors and recipients in each cell population (**Supplementary Tables S2**, **S3**), which revealed a shift across all major T-cell subtypes towards antigen-driven activation after transplantation. In this regard, our data reflect processes of activation, inflammation, and expansion that fit the clinical setting of alloHSCT, in which immune responses associated with infection, GvHD, and GvT occur (**Figure 2C**, see **Supplementary Figure S2B** for individual genes from these terms) (32, 33).

Top Ligand-Receptor-Interactions (LRIs) in an overrepresentation analysis (ORA) of differential cell-cell signaling between recipients and donors (34) (**Figure 2D**; **Supplementary Table S4**) reveal a striking upregulation of Signal Regulatory Protein-γ (SIRPG):CD47, which has been implicated in the context of T-cell activation and GvHD (35, 36). High scores were also observed for the interactions of S100A8/A9 with ITGB2, which have been linked to the induction of neutrophil chemotaxis and adhesion during inflammatory processes and immune response (37, 38), as well as the transmigration of leucocytes including T-cells in the context of GvHD (39, 40). Interestingly, salivary proteomic analysis in GvHD patients suggests S100A8 as a marker for GvHD activity (41). Also other top ranked interactions have been shown to mediate or balance allo-immune responses, such as CD72:SEMA4D (42), CD72:CD5 (43), HLA-DRB1/5:LAG3 (44) and MIF:CD74 (45).

More generally speaking, our cell-cell signaling network analysis for top LRIs underlines interactions between B- and T-cells that are relevant for the transplantation setting and that have been implicated in immune response and GvHD (**Figure 2E**).

### Clonal tracking of T-cells in alloHSCT using single-cell sequencing

**Figure 3:**
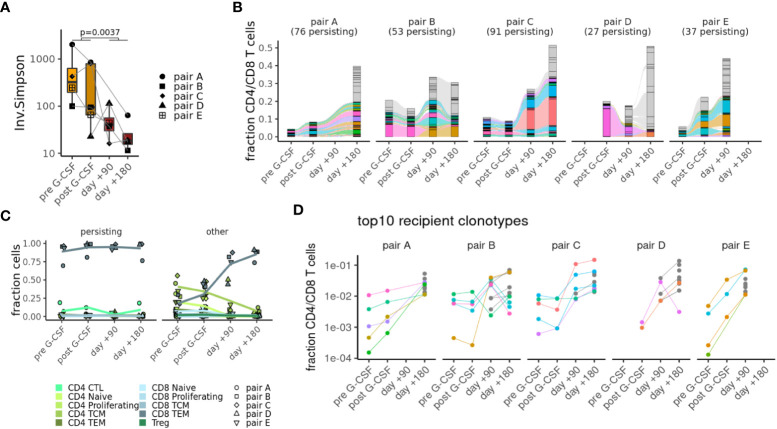
Clonal tracking of T-cells in alloHSCT. **(A)** Decrease in repertoire complexity from donors to recipients as measured by the inverse Simpson score. **(B)** Top clonotypes that are seen in both donor and recipient samples (colors) or else have > 1% abundance in any sample (gray). **(C)** Phenotypes of persisting clonotypes (seen in both donor and recipient samples) or other clonotypes at different time points. **(D)** Frequencies of top 10 recipient clonotypes shown at different time points for each pair.

To get a better understanding of clonal dynamics in alloHSCT, we focused our further analyses on single-cell TCR repertoires of the T-cell population. Comparing repertoire diversity between cells of donors and recipients, as measured by the inverse Simpson score (**Figure 3A**), we detected a significant decrease of the recipients’ repertoire diversity. Comparing repertoires between different samples (i.e, different time points as well as different individuals), we find the highest degree of clonal overlap between samples of the same individual and considerable overlap between matched donor and recipient samples as indicated by the Morisita score (see **Supplementary Figure S3A**). There is no overlap between samples of different pairs, suggesting that these results are not dominated by public clonotypes. Specifically, we were able to track between 27 and 91 clonotypes from donor to recipient up to six months posttransplant, using only TCRs with both α and β chain (**Figure 3B**; **Supplementary Figure 1C**). Notably, persisting αβT-cell clonotypes – as defined by their presence in the donor as well as the recipient of the same pair – expanded and represented at least 50% of the most abundant T-cell clonotypes in the recipients except in pair D, where T-cell clonotypes appeared to contract after transfer. This corresponds with clinical observations, as only this patient suffered a disease relapse after alloHSCT. When looking at T-cell phenotype attribution, persisting clonotypes were almost entirely annotated as CD8TEM (**Figure 3C**). In general, almost all cells in persisting αβT-cell clonotypes had the same phenotype across different samples, indicating that further differentiation is rare (**Supplementary Figure S3B**). When comparing our αβTCR information with available databases on antigen specificity, we found that only a fraction of our clonotypes expressed TCRs with known specificities such as CMV and EBV (**Supplementary Figure S3C**; **Supplementary Table S5**). Next, we tracked the fate of the ten most prevalent TCR clonotypes in the donor, as well as the origin of the top ten TCR clonotypes in the recipient. While top donor clonotypes did not show a clear bias towards expansion or contraction (**Supplementary Figure S3D**), most of the top recipient clonotypes underwent strong expansion over time (**Figure 3D**).

### Distinct transcriptional dynamics of persisting T cell clonotypes

**Figure 4:**
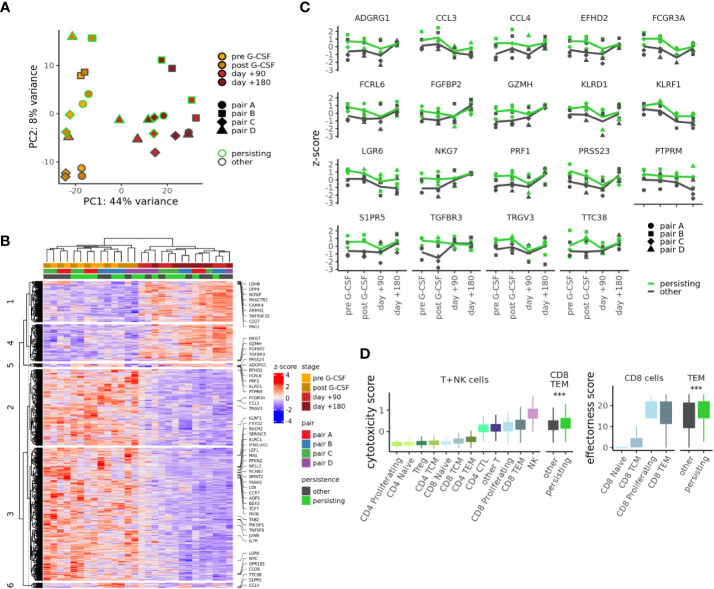
Distinct transcriptional dynamics of persisting T cell clonotypes. **(A)** PCA of pseudobulk gene expression in CD8TEM cells using 989 genes differentially expressed (adj. p-value < .01, abs. log2 fold change > 0.5) between donors and recipients or between persisting and other cells. **(B)** Gene expression heatmap for the genes used in **(A)**; 54 genes differentially expressed between persisting and other cells are highlighted. **(C)** Expression of selected genes differentially expressed between persisting and other CD8TEM cells at adjusted p-value < 0.01 and log2 fold change >0.5. **(D)** Cytotoxicity (left) and effectorness scores (right) for different T- or NK cell subpopulations. p-values from t-test, ***p < 0.001. CTL, cytotoxic T lymphocytes.

We next investigated the gene expression dynamics of persisting CD8TEM of samples from pairs A-D which passed our more stringent quality criteria (**Supplementary Figure S1A**). We performed PCA and unsupervised clustering of pseudobulk gene expression for the CD8TEM subpopulation at all collected time points using 953 genes differentially expressed in different comparisons: between donors and recipients and, within these groups, between persisting and other cells (**Figures 4A, B**). Both of these analyses demonstrate that gene expression changes are dominated by the donor-recipient difference. However, a unique transcriptional profile of persisting CD8TEM is connected to 54 genes (**Figure 4C** and **Supplementary Figure S4**) that are enriched in clusters 5 and 6. This profile is related to cytotoxicity and effectorness programs, as indicated by previously developed summary metrics (26, 27): persisting CD8TEM show significantly higher cytotoxicity scores (26) than other CD8TEM, reaching values closest to NK cells among all T-cell subsets (**Figure 4D**, left). Similarly, using an effectorness model originally developed for CD4^+^ T-cells (27) shows that persisting CD8TEM also exhibit higher effectorness than other CD8TEM (**Figure 4D**, right). Interestingly, the observed transcriptional differences are more pronounced in the donor samples, indicating that persisting CD8TEM clonotypes constitute a distinct and pre-existing donor T-cell population.

### Identifying persisting CD8TEM cells with cytotoxic features in the donor

**Figure 5:**
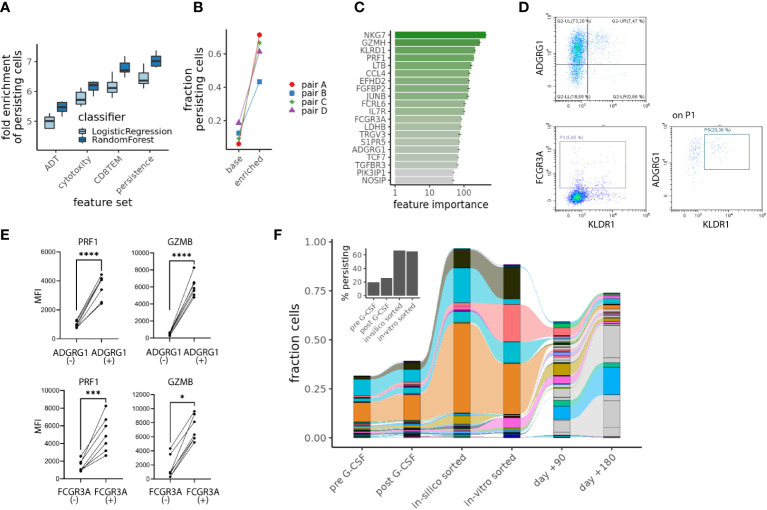
Identifying persisting CD8TEM cells with cytotoxic features in the donor. **(A)** Enrichment of persisting cells using different machine learning approaches and 4 different feature sets: antibody-derived tags (“ADT”), “Cytototoxicity” comprising cytotoxicity genes (26), “CD8TEM” comprising 50 markers for the CD8TEM population (24) and “persistence” including 54 genes differentially expressed in persisting CD8TEM. Boxes indicate interquartile range from 10fold cross-validation with a 75:25 train:test split across all ~26000 cells. **(B)** Results for the random forest model with 54 persistence genes when training on 3 donors and evaluating on the fourth. **(C)** Most informative features for the random forest model using 54 persistence genes. **(D)** Flow cytometric assessment of the surface marker ADGRG1, KLDR1 and FCGR3A on CD8TEM (% expression). **(E)** Flow cytometric assessment of the cytolytic molecules perforin (PRF1) and granzyme B (GZMB), linear graphs for five donors comparing CD8TEM with vs. without expression of ADGRG1 or FCGR3A. Illustrated are the Mean Fluorescence Intensities (MFI). P-values from t-test, *p < .05, ***p < .001, ****p < .0001 **(F)** Sorted ADGRG1+CD8TEM from donor of pair B were analyzed by TCRβ bulk sequencing and clonotypes with TCRβ chains, which overlapped with our persisting T cell clonotypes from the scRNAseq analysis are shown. Inset shows the percentage of persisting cells in donor samples.

To address this further, we asked to which extent this persisting T-cell population could be identified and isolated already in the graft. Indeed, we observed a systematic shift between persisting and other CD8TEM cells in donors when projecting single cells into the PCA of **Figure 5A** (**Supplementary Figure S5A**). Hence, we first performed machine learning in order to enrich persisting CD8TEM from CD4^+^ or CD8^+^ T-cells, evaluating the performance of two different classifiers and four different feature sets: 10 surface antigens from our CITEseq data (**Supplementary Figure S1B**), 12 cytotoxicity genes from literature (26), the top 50 markers for the CD8TEM population of the PBMC reference we used (24) or the 54 genes differentially expressed in persisting CD8TEM. A random forest model with the 54 persistence genes showed optimal performance, reaching a ~7-fold enrichment of persisting CD8TEM (**Figure 5A**). Training this model on three donors and evaluating on the fourth, we similarly found that the abundance of persisting CD8TEM could be increased by a factor 3-12 from the baseline of 6-19% to values between 43-71% (**Figure 5B**). The most informative features for this classifier include expected cytotoxicity and effectorness genes such as NKG7 (encoding for Natural Killer Cell Granule Protein 7) and GZMH (encoding for Granzyme H) as well as surface markers Adhesion G Protein-Coupled Receptor G1 (ADGRG1=GPR56), Killer Cell Lectin Like Receptor D1 (KLRD1=CD94) and Fc Gamma Receptor IIIa (FCGR3A=CD16A) (**Figure 5C** **)**. Using flow cytometry, we could readily detect subpopulations with substantial protein expression of ADGRG1, KLRD1 and FCGR3A in CD8TEM of five additional healthy donors that were not included in our scRNAseq experiments (**Figure 5D**). Comparing ADGRG1^+^ or FCGR3A^+^ CD8TEM populations against ADGRG1^-^ or FCGR3A^-^ controls, respectively, we in fact measured higher cytotoxic functionality by means of significantly increased expression of perforin (PRF1) and granzyme B (GZMB) (**Figure 5E**).

In a proof-of-principle experiment, we finally sorted ADGRG1^+^ CD8TEM from one of our alloHSCT donors (donor B from pair B) and performed TCRβ bulk sequencing. When comparing these TCRβ bulk sequencing results of cells from donor B with the single-cell TCR sequencing results of cells from donor B, we detected 54 TCRβ chains that were present in both the single-cell and the bulk TCR data. 26 of these 54 TCRβ chains were among the persisting T-cell clonotypes (defined based on single-cell TCR sequencing as described above, see also **Supplementary Figure S5B**). We obtained a substantial enrichment of persisting cells by this “*in-vitro*” sort (**Figure 5F**), by a comparable factor to the “*in-silico*” sorting from **Figure 5B**.

These results confirm that it is indeed possible to enrich CD8TEM with enhanced cytotoxicity and effectorness from the donor graft that show expansion and long-term persistence after transfer to the recipient.

## Discussion

Donor T-cells mediate GvT and are essential for immune defense in early immune reconstitution, and their effectiveness therefore determines the overall success of alloHSCT. To gain highly resolved information on persisting T-cell clonotypes and the associated gene expression patterns, we studied alloHSCT donor-recipient pairs for up to 180 days after transplantation. Looking first at overall immune reconstitution in the recipients, CD8^+^ T-cells dominated the T-cell compartment post alloHSCT. This is consistent with extensive data on immune reconstitution after alloHSCT showing a predominance of CD8^+^ T-cells resulting from lymphopenia-induced homeostatic proliferation and antigen activation (14, 46). Clinical studies equally confirm the relevance of CD8^+^ T-cells in the alloHSCT setting (47, 48).

Next, we looked at changes between donors and recipients to gain a better understanding of shifts in cellular immunity between healthy donors and transplant recipients on single cell level. Top LRIs in an ORA of differential cell-cell signaling between recipients and donors revealed an upregulation of interactions mediating or regulating allo-immune responses. Antibody blocking experiments support roles in GvHD for SIRPG, LAG3 and CD74. Antibody blockade of SIRPG impaired IFNγ secretion by activated T-cells and hindered SIRPG:CD47 interaction resulting in significantly delayed onset of GvHD and impaired donor chimerism (35, 36). The interaction of SIRPG and CD47 also has been shown to play a key role in transendothelial migration of T-cells under shear flow conditions (49) and promotes antigen-specific T-cell proliferation and T-cell costimulation (36). Lag-3 as an important regulatory molecule involved in alloreactive T-cell proliferation and activation after bone marrow transplantation (44) and blockade of the lymphocyte-activated gene-3 (LAG-3) signaling prevented murine GvHD (50). CD74 is widely expressed in antigen-presenting cells such as B-cells, and GvHD could be prevented by anti-CD74 antagonistic antibodies (51). Expression of the interaction partner MIF has been shown to control functional activation of CD74 (45) and is upregulated in alloHSCT. The role for SEMA4D in the alloreaction by modulating T-cell-APC interaction is supported by knock-out (KO) T-cell experiments demonstrating that murine recipients of SEMA4D KO T-cells exhibit reduced mortality and GvHD while GvT is preserved (42). Overall, we observe interactions driven by alloreactivity in our posttransplant samples, which are consistent with other studies in similar settings and might be candidates to mitigate GvHD while maintaining GvT. Larger cohorts would allow more detailed analyses with respect to differences in clinical characteristics between individual pairs.

Combining immune profiling with scRNAseq data, we identified a specific peripheral CD8TEM subset in the context of alloHSCT by tracking T-cell receptor sequences from the donor to the recipient. Even though single-cell immune profiling samples only a relatively small fraction of the T-cell receptor sequence repertoire and we therefore likely undersample persisting CD8TEM clonotypes, we still observed a distinct gene expression profile when comparing to CD8TEM clonotypes that appeared exclusively at one time point. We were thus able to attribute a specific molecular phenotype to these persisting cells that enabled their identification already in the graft *via* protein surface markers. Some of the upregulated genes in persisting CD8TEM were associated with NK functions such as ADGRG1, FCGR3A and KLRD1. ADGRG1 (=G-protein coupled receptor 56, GPR56) is expressed on human circulating NK-cells and CD8^+^/CD4^+^ CTL (52, 53). Expression of FCGR3A, synonyme for CD16, on CD8^+^ T-cells has been associated with NK cell-like functional properties (54), and interestingly, high expression of KLRD1 on NK- and CD8 T-cells has been correlated with lower level of apoptosis and maintenance of these cells (55). Accordingly, CD8TEM score right alongside NK-cells in metrics for cytotoxicity and effectorness (26, 27).

A related study by Sheih et al. evaluated *in vivo* performance of chimeric antigen receptor (CAR) T-cells by scRNAseq and clonal tracking. Transcriptionally distinct clusters of CAR T-cells in the infusion products of four patients characterized by specific expression of genes associated with T-cell activation, cytotoxicity, mitochondrial functions, and cell cycle, were found to yield different contributions to the CAR T-cell pool in the blood at later time points after infusion (56). As in our study, the longest persisting clonotypes exhibited elevated expression of cytotoxicity genes such as GZMH and NKG7. This suggests that our findings may be relevant for adoptive T-cell therapy in general.

Another study explored the influence of different CAR signaling domains and their effect on the gene expression of T-cells, suggesting that this knowledge could support the production of more precise CARs as the differences are known already before infusion (57). Similarly, in our clinical context of inter-individual cell transfer, we observed a distinct transcriptional profile of persisting CD8TEM (compared to other CD8TEM) already in the donor, independent of G-CSF mobilization, and prior to cell transfer to the patient. Thus, we hypothesize that persisting CD8TEM clonotypes constitute a distinct and pre-existing donor T-cell population that could be identifiable in any given cell sample. Importantly, since surface markers are part of the identified gene signature, the persisting CD8TEM subset could be selected by flow cytometry. Due to our experimental approach, we are unable to make inferences about the biological role of the identified cells in alloHSCT, i.e., we cannot assess whether these clones primarily support GvT, infection defense or GvHD. However, the identification of T-cell attributes in the donor that are connected to persistence of T-cell clones is a step towards more precise donor graft composition strategies.

Taken together, we examined the *in vivo* behavior of individual TCR donor clonotypes. Naturally, a number of other factors besides cell intrinsic properties might impact the expansion and persistence of donor T-cells. However, our results contribute to a deeper understanding of graft composition in alloHSCT and may be an essential building block for future studies addressing personalized graft manipulation strategies, as we identified a persistent CD8TEM subset that could potentially be selected prior to transplantation if further research in the context of antigen specificities of interest confirms beneficial clinical effects of this subset.

## Data availability statement

The datasets presented in this study are deposited in NCBI's Gene Expression Omnibus, accession number GSE222633. Analysis code is available at https://github.com/bihealth/obermayer_et_al_tcell_persistence.

## Ethics statement

The studies involving human participants were reviewed and approved by Ethics committee of Charité Universitätsmedizin Berlin. The patients/participants provided their written informed consent to participate in this study.

## Author contributions

BO conceptualized and performed all bioinformatic analyses and wrote the manuscript. LK planned and performed experiments, supported bioinformatic analysis and wrote the manuscript. TC supported experimental planning and execution. MF supported study conceptualizing, experimental planning, and data analysis. I-WB, LV, KM, CT-B, OP, and LB supported patient selection, sample acquisition, clinical data collection and analysis. SL supported sample acquisition, sample processing, clinical data collection and analysis. LH and LL performed the FACS sorting. US and NB performed the TCRβ bulk sequencing and analysis. SH and DB supported bioinformatic analyses and data interpretation. FW performed part of the experiments. FW and I-KN conceptualized the study, supported patient selection and inclusion, planned and oversaw experiments, participated in bioinformatic analyses, and wrote the manuscript. All authors contributed to the article and approved the submitted version.
