# Supplementary material for: Single-cell clonal tracking of persistent T-cells in allogeneic hematopoietic stem cell transplantation
Source: Front Immunol. 2023 Feb 10;14:1114368. doi: 10.3389/fimmu.2023.1114368 (PMC9969884; doi:10.3389/fimmu.2023.1114368)
Supplement: Supplementary file 1 [file DataSheet_1.docx]

**Table S1. Clinical details for the pairs A-E.**


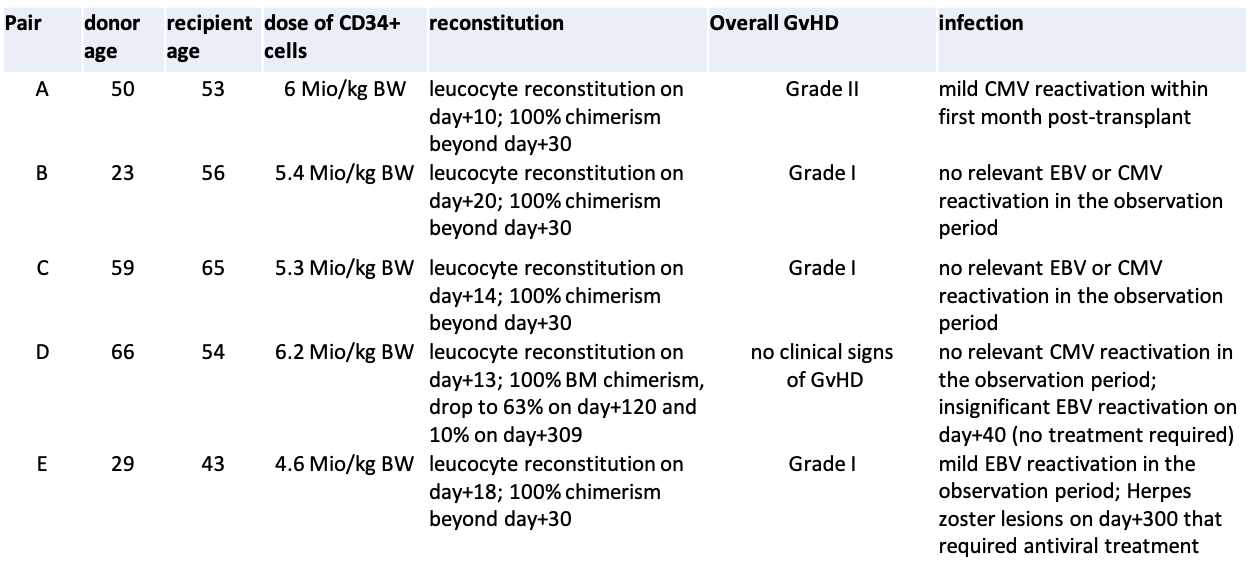


**Donor A** was 50 years of age at the time of mobilization and apheresis. The transplant included 6x10^6^ CD34+ cells per kg bodyweight of the recipient. Recipient A (53 years) showed leukocyte reconstitution on day +10 post transplantation and bone marrow chimerism was 100% beyond day +30. **Recipient A** developed overall acute GvHD (aGvHD) grade II with and onset of grade III skin aGvHD on day +27 and mild CMV reactivation within first month posttransplant.

**Donor B** was 23 years of age at the time of mobilization and apheresis. The transplant included 5.4x10^6^ CD34+ cells per kg bodyweight of the recipient. **Recipient B** (56 years) showed leukocyte reconstitution on day +20 post transplantation and bone marrow chimerism was 100% beyond day +30. Recipient B developed overall aGvHD grade I with onset of grade I skin aGvHD on day +12 and moderate chronic GvHD mainly of the skin with onset on day +240. There was no relevant EBV or CMV reactivation in the observation period.

**Donor C** was 59 years of age at the time of mobilization and apheresis. The transplant included 5.3x10^6^ CD34+ cells per kg bodyweight of the recipient. **Recipient C** (65 years) showed leukocyte reconstitution on day +14 post transplantation and bone marrow chimerism was 100% beyond day +30. Recipient C developed overall aGvHD grade I with and onset of grade I skin GvHD on day +20. There was no relevant EBV or CMV reactivation in the observation period.

**Donor D** was 66 years of age at the time of mobilization and apheresis. The transplant included 6.2x10^6^ CD34+ cells per kg bodyweight of the recipient. **Recipient D** (54 years) showed leukocyte reconstitution on day +13 post transplantation and bone marrow chimerism was 100% up until day +120 when chimerism fell to 63%. A total of five donor lymphocyte infusions were administered until day +308. Chimersim was 10% on day +309. Recipient C did not show signs of aGvHD. There was no relevant CMV reactivation in the observation period. Recipient C developed minor EBV reactivation on day +40 which did not require treatment.

**Donor E** was 29 years of age at the time of mobilization and apheresis. The transplant included 4.6x10^6^ CD34+ cells per kg bodyweight of the recipient. **Recipient E** (43 years) showed leukocyte reconstitution on day +18 post transplantation and bone marrow chimerism was 100% beyond day +30. Recipient F developed overall aGvHD grade I with and onset of grade I skin GvHD on day +29. Mild EBV reactivation in the observation period. On day +300 Recipient E showed Herpes zoster lesions that required antiviral treatment.

**Suppl. Figure S1. (A)** QC metrics for each sample. Violin and box plots show counts of UMIs per cell (nCount_RNA), counts of genes per cell (nFeature_RNA), mitochondrial content (pct.mito) and prediction score for label transfer from the PBMC reference (predicted.celltype.l1.score). **(B)** Enrichment of antibody-derived tags of CITEseq for canonical markers in the associated immune populations. **(C)** Cell type composition of the different samples. TCM = central memory T-cells; TEM = effector memory T-cells; DC = dendritic cells; Mono = monocytes; NK = natural killer cells; Treg = regulatory T-cells; pre = pre G-CSF; post = post G-CSF; d90 = day +90 post transplantation; d180 = day +180 post transplantation.

**Suppl. Figure S2. (A)** UMAP embedding showing clonotype abundance (hyperexpanded: > 1%, large: > 0.1%, medium: > 0.01%, small <= 0.01%). **(B)** Top genes differentially expressed between recipients and donors for each cell type and associated with pathways shown in Figure 2C

**Suppl. Figure S3. (A)** Clonal overlap (Morisita score) between different samples. **(B)** Fraction of cells with the majority phenotype across different samples for each persisting clonotype from Figure 3B. **(C)** Frequency of clonotypes with known CMV or EBV specificity. **(D)** Frequencies of top donor clonotypes are shown at different time points for each pair.

**Suppl. Figure S4.** Selected genes differentially expressed between persisting and other CD8TEM cells at adjusted p-value < 0.01 and abs. log2 fold change > 0.5. Cluster membership is indicated.

**Suppl. Figure S5. (A)** Single CD8TEM cells projected into the PCA of Fig. 4A. **(B)** TCRβ bulk sequencing of sorted ADGRG1+CD8TEM from one alloHSCT donor (pair B). 54 TCRβ chains both in single-cell and bulk TCR data were detected, 26 of which were among the persistent T cell clonotypes.


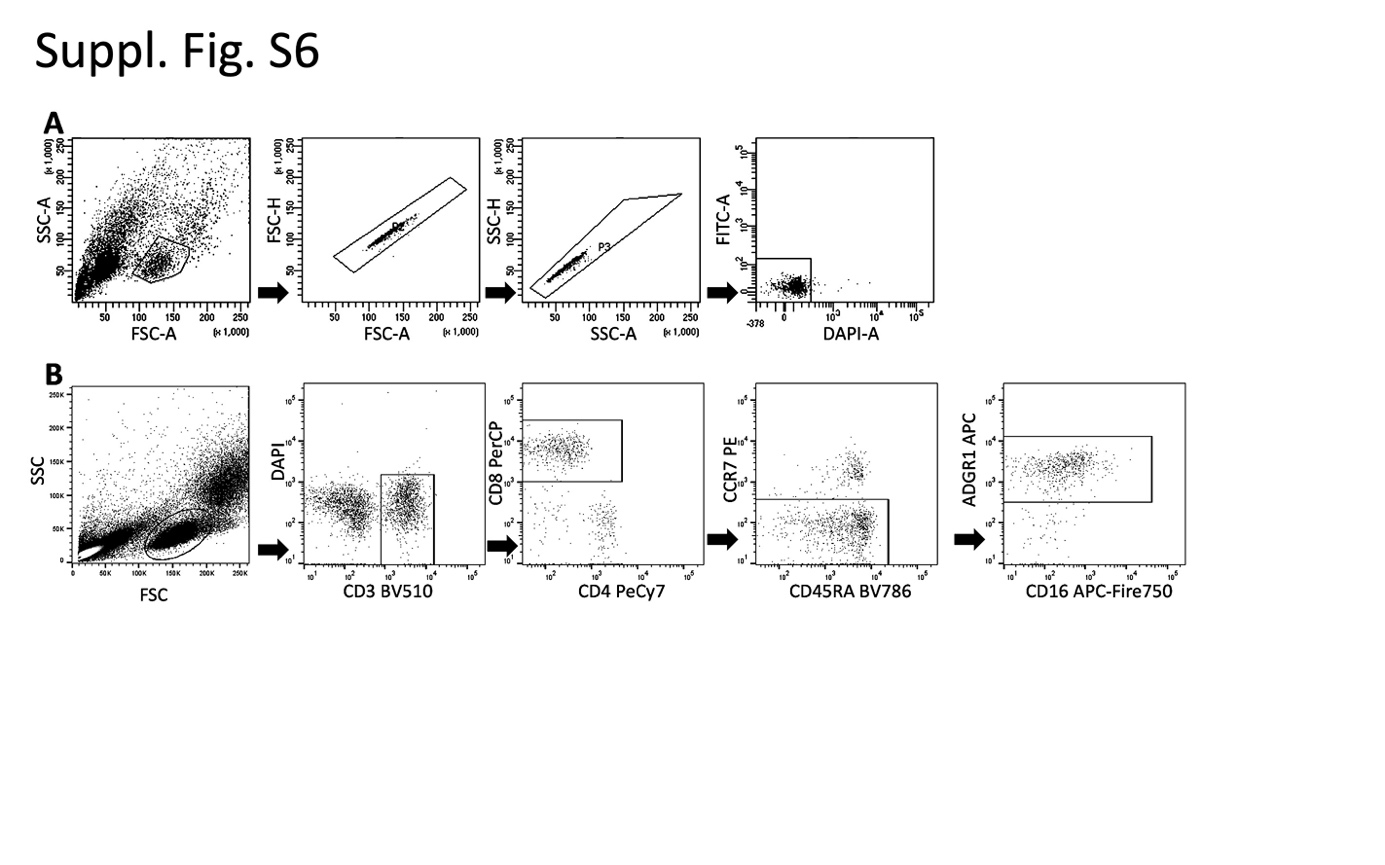


**Suppl. Figure S6. (A)** Representative example of sorting strategy for all single-cell sequencing experiments. **(B)** Representative example of sorting strategy for functional experiments and TCRβ bulk sequencing analysis, both shown in Figure 5 of the manuscript.
